# Exploring the sorting of patients in community health centres across Gauteng Province, South Africa

**DOI:** 10.1186/s12875-018-0899-y

**Published:** 2019-01-07

**Authors:** B. A. Stott, S. Moosa

**Affiliations:** Department of Family Medicine, Faculty of Health Sciences, University of the Witwatersrand, Johannesburg, South Africa

**Keywords:** Primary health care, Outpatient, Non-emergency, Triage, Sorting

## Abstract

**Background:**

Primary health care worldwide faces large numbers of patients daily. Poor waiting times, low patient satisfaction and staff burnout are some problems facing such facilities. Limited research has been done on sorting patients in non-emergency settings in Africa. This research looked at community health centres (CHCs) in Gauteng Province, South Africa where queues appear to be poorly managed and patients waiting for hours. This study explores the views of clinicians in CHCs across Gauteng on sorting systems in the non-emergency ambulatory setting.

**Methods:**

The qualitative study design used one-to-one, in-depth interviews of purposively selected doctors. Interviews were conducted in English, with open-ended exploratory questions. Interviews were recorded, transcribed, anonymised and checked by interviewees later. Data collection and analysis stopped with information saturation. The co-author supervised and cross-checked the process. A thematic framework was developed by both authors, before final thematic coding of all transcripts was undertaken by the principal author. This analysis was based on the thematic framework approach.

**Results:**

Twelve primary health care (PHC) doctors with experience in patient sorting, from health districts across Gauteng, were interviewed. Two themes were identified, two major themes, namely Systems Implemented and Innovative Suggestions, and Factors Affecting Triage. Systems Implemented included those using vital signs, sorting by specialties, and using the Integrated Management of Childhood Illnesses approach. Systems Implemented also included doctor - nurse triage, first come first serve, eyeball triage and sorting based on main complaint. Innovative Suggestions, such as triage room treatment and investigations, telephone triage, longer clinic hours and a booking system emerged. There were three Factors Affecting Triage: Management Factor, including general management issues, equipment, documentation, infrastructure, protocol, and uniformity; and Staff Factor, including general staffing issues education and teamwork; and Patient Factor.

**Conclusion:**

Developing a functional triage protocol with innovative systems for Gauteng is important. Findings from this study can guide the development of a functional triage system in the primary health care non-emergency outpatient setting of Gauteng’s CHCs. The Emergency Triage, Assessment and Treatment (ETAT) tool, modified for adult and non-clinician use, could help this. However, addressing management, staff and patient factors must be integral.

## Background

Primary health care facilities worldwide, face increasing numbers of patients daily. Due to this, long waiting times, low patient satisfaction, and staff burnout are some of the problems facing such facilities. Limited research has been done on sorting of patients in non-emergency settings in Africa. It is evident from the literature that many different sorting or triage methods for this setting have been attempted internationally, possibly highlighting the challenging nature of this environment [1–3].

Triage involves sorting large numbers of patients into categories according to the urgency of their needs [4]. The concept of sorting patients in South Africa started with the Cape Triage Score, which uses physiological parameters and discriminators and groups patients into trauma codes [5]. The South African Triage Score (SATS), validated for use in emergency departments, was then rolled out and spread to six other sub-Saharan countries [6]. Since then its validity, interrater and intra-rater reliability had been proven for low-resource countries other than South Africa [7, 8]. Unfortunately, these findings apply only to emergency departments settings. A systematic review of emergency department triage scales, found that there is generally poor evidence for or against patient sorting and that there is poor agreement between observers [9]. The study also showed that the ability of a single vital sign to predict mortality was limited. The time involved in measuring the necessary parameters is also lengthy and this may not be viable in a busy primary health care outpatient department [10]. There are questions about the limited reproducibility of vital signs, as well as sorting systems that do not use vital signs [11, 12].

Limited research has been done on the sorting of patients in the non-emergency outpatient setting. These settings have high patient volumes, low staff numbers and inadequate equipment, which limit the use of the SATS. Other South African attempts at sorting patients to shorten waiting times in this setting is the use of a fast queue system. Unfortunately, the influx of patients has resulted in this fast queue system being ineffective [13]. If demand and capacity are balanced, waiting time and number of visits would decrease, and the need for patient sorting would most likely not exist [14]. Some methods tried internationally include self-triage with a complaint tick-sheet which reduced waiting times significantly and allowed urgent cases to be prioritized [15]; an appointment system where certain time slots were allocated for the un-booked emergency patients [16]; telephone triage [17]; “see and treat” [18]. In Africa there is only a study on midwife-led triage for an obstetric unit in Ghana [19].

Gauteng community health centres (CHCs) currently have no standardised system for sorting patients in the outpatient non-emergency setting. The Ideal Clinic guidelines recommend: three hours as a maximum waiting time; a dedicated area for monitoring vital signs; and a process that prioritises high risk patients [20]. There are anecdotal reports of clinicians using different systems in crowded CHCs to sort walk-in patients. These have not been documented. The aim of this study was to explore the views of clinicians in CHCs across Gauteng on sorting systems to manage walk-in patients in this setting.

## Methods

This qualitative study was designed to use in-depth interviews of purposively selected doctors involved in the implementation of sorting systems in non-emergency outpatient settings at CHCs in Gauteng health districts (Johannesburg; Ekurhuleni; Tshwane; Sedibeng; and West Rand). The District Family Physician in each health district was approached to obtain a list of such clinicians. This was expanded with snowball sampling to obtain a diversity in district location, age, gender, and years in practice to ensure “information-richness”. A pilot study was undertaken prior to embarking on formal data collection. The interview guide was not changed after the pilot study.

The one-to-one interviews, lasting approximately 30 min, were conducted in English by the principal author at the workplace of the participant. It was digitally recorded with two digital recorders and facilitated using a short list of open-ended, exploratory questions as an interview guide. The interview guide explored the clinicians views on sorting systems to manage walk-in patients, their reasons for choosing a particular sorting method, their views on factors enabling or inhibiting attempted systems. It also gave the clinician the opportunity to add any additional information. Interviews were conducted over a two-month period (May–June 2017). Informed consent was provided by participants in writing. The principal author transcribed all digital recordings verbatim during the interview phase (after each interview) and these were checked by the co-author. The transcripts were sent back to participants for review (member checking) to ensure trustworthiness and accuracy.

The data has been kept anonymous and only the principal author knows which transcript belongs to which participant. The principal author and co-author analysed the anonymized data using the framework approach [21]. The principal author and co-author familiarized themselves with the data. A thematic framework was developed together, before final thematic coding was done by the principal author. The themes were further analysed to assess relationships between themes. Data collection and analysis stopped with information saturation. No repeat interviews were done. The co-author supervised and cross-checked the process. Quotations were used from different participants to ensure trustworthiness and transparency. The COREQ guideline was used to review the validity of the method [22].

## Results

Twelve diverse participants from all health districts in Gauteng were interviewed (See Table 1).

**Table 1:** Participant Demographics

PARTICIPANT	A	B	C	D	E	F	G	H	I	J	K	L
DISTRICT	JHB^a^	JHB^a^	TSH^b^	TSH^b^	TSH^b^	JHB^a^	JHB^a^	JHB^a^	WR^c^	SED^d^	SED^d^	EKH^e^
YEARS OF EXPERIENCE	10	12	24	16	12	10	15	12	17	8	34	20

(^a^ Johannesburg Metro; ^b^ Tshwane; ^c^ West Rand; ^d^ Sedibeng; ^e^ Ekurhuleni)

**Fig. 1:**
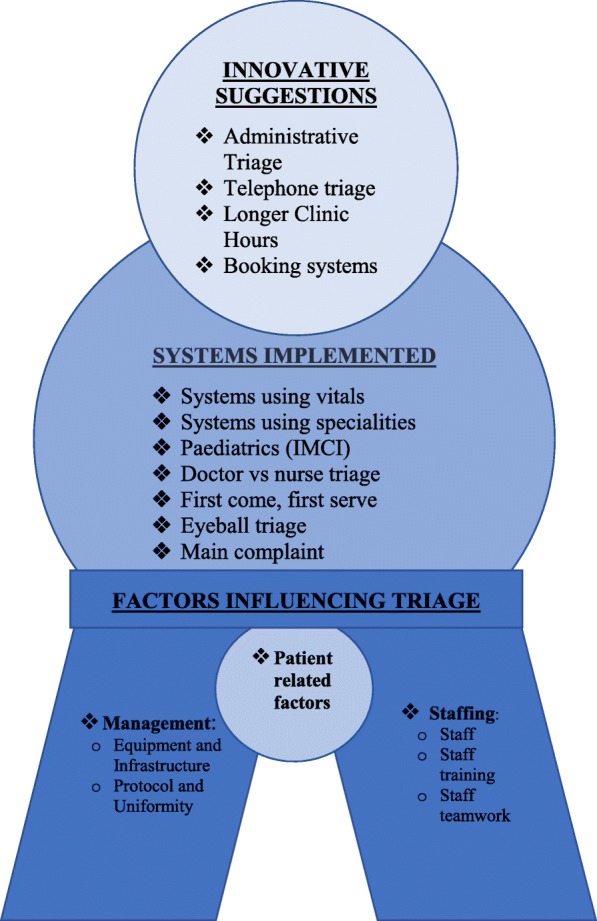
Overall Themes

Two major themes emerged from the data: A) Systems Implemented and Innovative Suggestions, and B) Factors Influencing Triage. (See Fig. 1).

A) **Systems Implemented and Innovative Suggestions**

Systems implemented emerged as a major theme. Participants described implementing a number of systems. Some innovative suggestions emerged as well:

### Systems using vitals

Some participants described implementing the South African Triage Score, with some modifications. They did get vitals by enrolled nurses, albeit not completely. Patients were then sorted into those with problems and those for repeat scripts or blood investigations, sometimes the elderly and children were separated if they looked sick.

*‘I came up with a triage system where you use a discriminator, you first use the vital signs. Is the patient mobile? Is the patient eating? the blood pressure, the temperature, the GCS (Glasgow Coma Scale) and then you use a discriminator and you score accordingly.’ (L)*

### Systems using specialty

Some participants reported implementing a sorting system using sections that separated patients either into acute or chronic patients, or based on a specialty or type of service (e.g., gynaecology, paediatrics, HIV, TB).

*‘What we decided to do is to sort them by chronic (or) minor complaints. Then we divide by specialties. Pregnant woman and children have their own way’ (K)*

### Systems for paediatrics

All facilities had a separate paediatric section. Children were sorted into those going for well-baby clinic and acute patients requiring Integrated Management of Childhood Illnesses (IMCI). IMCI is run by trained nurses:

*‘Paediatrics is actually seen in a separate setting with IMCI and acute and chronic care. If it is an emergency, they come straight to casualty’ (E)*

Many spoke about the importance of rapid paediatric sorting due to the risk of faster deterioration in this age group:

*‘Children will need a special triage area where they are triaged quickly because they are likely to get into trouble if their condition changes’ (D)*

From the interviews done, it seems as though the paediatric sections are running well thanks to the IMCI program and adequate training.

### First-come first-serve system

Many facilities still use the first-come first-serve system:

*‘Basically, patients are sorted out as they come. They get first come, first served. So, they get their file, they get their vitals taken and then they are either seen by a professional nurse or a doctor’ (I)*

### Systems for doctor’s vs nurses

While this was a less commonly occurring system, some facilities described a process of separating patients into those suitable for nurses and those suitable for doctors. The importance for distinguishing junior from senior doctors was also raised:

*‘Separate the patients which are suitable to be seen by the PHC nurses and others by the doctors’ (B)*

*‘We have senior doctors and we have junior doctors looking at patients, and you wouldn’t want a serious patient going to a junior doctor’ (G)*

### Eyeball triage system

This is done in some facilities by a variety of people. Patients who appear ill are removed from the line regardless of their vital signs.

### Main complaint system

Some facilities sort patients based on their main complaint and decide whether vitals are done or not:

*‘We didn’t have any vitals per se but we would have a doctor there who would triage clinically based on complaint. It was the most senior person triaging because obviously, you want the most experienced people to be triaging.’ (F)*

### Administrative triage system

It was suggested that some patients could be managed from the triage room where, for example, stat doses of medications could be given for hypertensives so that they are covered while waiting to be seen. X-ray forms could also be completed in the room.

### Telephone triage system

Telephone sorting was suggested in two forms: One, where patients call in and it is decided over the phone if they need to come immediately, in a few days or whether they need a home visit by the community health workers. Two, where nursing staff in smaller referring clinics can call a doctor-on-call for assistance:

*‘Get patients to call in, lets triage them according to that process: What are you feeling? Advise them, get a community health worker to visit them, see what their level of disability is, what they are coming for, and this could all be done by telephone triage’ (A)*

*‘The doctor leaves his number with that clinic, then they know that he is in the area or somewhere, you can phone your doctor if you have a problem anytime in the week’ (I)*

### Longer hours and booking system

Longer clinic hours were suggested to help deal with the large numbers of non-emergency patients. And a booking system with allocated times was suggested for the chronic patients to help control the number of patients coming on a day, as well as reducing the waiting times. This was tried and failed in some facilities.

B) **Factors Influencing Triage Systems**

Participants described two major factors that influenced the implementation of any sorting systems: management and staffing. Patient factors emerged as a smaller yet related issue.

### Management

There seemed to be no accountability or consequences, so staff get away with doing whatever they like. This makes implementation and sustainability of a system challenging if not impossible. Doctors have very little say and the system seems quite abusive towards doctors as management positions are occupied by nurses. Managers who consider all opinions; who are committed to the system being implemented; and have a more efficient management style

*‘It was done for a day or two and after that the system collapses because you don’t watch the system. The manager should be watching the system’(D)*

*‘Doctors don’t control the environment’ (A)*

*‘It has to start from management level and they have to identify (given our resources, given our staff) what will work’ (E)*

The lack of functioning equipment was raised as an inhibiting factor. Sorting was further challenged by inappropriate infrastructure, space and facilities such as toilets at our CHCs.

*‘(We) battle to find machines that work’ (A)*

*‘Infrastructure is one of the reasons as to why we have a non-functional triage system and not a quality triage system running because our triage is literally done in the corner of the reception where the patients come and get their files’(E)*

The need for documentation or a template to guide those working in the sorting room was raised. The lack of a resource-appropriate protocol prevents standardization and creates opportunities for resistance. The importance of a uniform system across all facilities was raised:

*‘I think what could really help would be to develop some kind of clear protocol.’ (A)*

*‘Once it is protocol that this needs to be done, then nobody is going to be against it’ (J)*

### Staff

Several issues around staffing emerged. The lack of teamwork and poor relationship between doctors and nurses was repetitively raised. The issue around teamwork and buy-in emerged as an important factor for an effective system. There seemed to be resistance from staff members and lack of commitment to implemented systems. There were mixed feelings from many participants as to who should do the sorting. Some felt it should be the nurses whilst others felt that it should be the most senior doctor clinician. They raised some issues with the nurse’s ability to do sorting. Interpretation of results by junior staff is difficult and it was felt that it should therefore be a senior, experienced staff member working in the sorting area:

*‘Personnel is a barrier. We cannot spare a doctor, we cannot spare a professional nurse to do proper triaging.’ (E)*

*‘They (clinicians) felt it was cumbersome. They also felt it was challenging. They also felt, in my opinion, that it would open up or expose things but they failed to understand the underlying reason for it, that it would result in better patient care and improve the quality of care so that nobody is missed’ (J)*

*‘Even the cold patients, the ones the doctor doesn’t really have to see, end up being seen by doctors because they (nurses) do it intentionally’ (J)*

Respondents describe the importance of buy-in and teamwork by all staff members and management. For the system to work it requires commitment by all and not just a single person. It is also important that everyone understands the system and its benefits to staff and patients**:**

*‘All of us must come on board. The clerk, the staff nurse, the sisters, the doctors, the management, the queue marshal’ (C)*

*‘If we help each other the health care will be better and seeing the patients throughout the day will be easier’ (B)*

The lack of staff training around triage and sorting of patients was also highlighted.

*‘Getting personnel trained is also a barrier. Most of our staff is not trained in how to adequately manage the triage area’(E)*

The lack of adequate numbers of staff employed, the high turnover of staff and the lack of senior staff at facilities were issues raised;

*‘The changing of staff, the turnaround. New people, they come, they resign, they change, then it becomes a problem. Every time we have to re-educate’ (K)*

### Patient

Patient related factors were raised by some clinicians interviewed. Large patient volumes affect patient flow making sorting challenging. The lack of patient education with regards to sorting results in impatience, excuses and complaints. Educating patients about the sorting system and continuously communicating with them would enable a better system:

*‘Patient education is very important because they might be sitting there, many of them …. have been there since 7 or 8 ‘o clock. As soon as an emergency comes they are not keen to let the emergency go through’ (C)*

## Discussion

The results described sorting systems that participants had implemented using vitals for South Africa Triage Scores (SATS), specialties (including paediatrics), allocating patients by suitability for doctors vs. nurses, sorting by complaints and simple first-come first served to manage busy outpatient departments in certain CHCs. There were some innovative suggestions as well: administrative triage rooms, triage by telephone, longer clinic hours and a booking system. They also described two major factors influencing the implementation of sorting systems: management and staff. Management factors included equipment and infrastructure as well as documentation and standardized protocols for sorting. Staffing factors included appropriate staff members doing sorting and their training, as well as the buy-in of all staff into a sorting system. Patient factors were viewed as a minor issue by the participant. Patients were not interviewed in this study.

Modern day sorting systems need to be able to accommodate a full spectrum of clinical conditions, from minor illness and injury to critical conditions, from paediatrics to geriatrics [23]. Results of this study indicate that while many different sorting systems have been implemented across Gauteng’s CHCs in the non-emergency outpatient setting, none of these attempts reported by interviewees have been an outright success.

Attempts by clinicians in Gauteng’s CHCs at implementing the SATS have been unsuccessful in this setting. This corresponds with challenges around vital sign recording [9], equipment availability, staff requirements to ensure rapid patient flow, time available per patient [10], reproducibility [9] and actual need [11]. The use of IMCI was reported to be functioning well in the primary health care paediatrics setting. Sorting patients based on specialty still leaves the undifferentiated patient lurking, and further sorting within each specialty would still be required. The literature shows that triaging to a specialty significantly increases triage times [18]. It is easy to default to first-come, first served in a fragmented specialty-based approach common in Gauteng CHCs. Many CHCs struggle with well-functioning triage rooms and constant disputes about who should drive the process, doctors or nurses, as well as disputes in deciding which patients should go to doctors and which to nurses. The urge to push queues often subverts the clinical process [24].

Suggested intervention such as telephone sorting removes visual cues, which may be important in the sorting process. Triage designation between telephone and in-person triage have not been found to be equivalent [25]. The safety of telephone sorting is still questionable and unlikely with the lack of the resource of telephones at CHCs [17]. A “see and treat” system which treated simple conditions in the triage room, prolonged the waiting time for some while decreasing it for others [18, 26]. There needs to be more research to show that sorting by senior experienced staff, including using ‘eyeball’ triage, would be more efficient than the current system to convince managers to implement such an approach. Longer clinic hours would need to be balanced with staff availability. Resource management is a key factor in success.

The two main factors influencing the partial success or failure of triage systems were reported to be issues around management and staffing. Challenges with management capacity and doctor-nurse teamwork in the South African clinic setting have been found previously [27]. It is necessary for management to monitor and take responsibility for the system, which would also help instill a sense of trust [28]. With adequate staffing, there is a need for triage personnel to rely on their education and experience, while integrating patient’s clinical information and care environment and avoiding bias [17]. This highlights the need for experienced personnel to run the triage. Training staff is essential to help build team capacity [29]. Training produces better agreement between staff [30], and better understanding of the system and thus ownership of the problem [31].

While the issue around inadequate staff numbers was raised, action research demonstrated that simple changes to daily activities like assigning staff to different tasks throughout the day, having a short morning staff meeting, shifting non-urgent tasks to less busy hours of the day, and colour coding files, significantly reduced the mean waiting times of patients, incorporated a form of triage, and was a more efficient system with staff numbers at that time [32]. With the current challenges around employment it is essential to find solutions that utilize current staff members efficiently and maximize their contributions whilst also making sure they do not reach a point of burnout. Equipment and adequate infrastructure are needed for any system to function.

A standardised validated tool as well as a protocol can be developed to assist patient sorting in this setting. This is consistent with recommendations internationally, especially to avoid the medico-legal challenges plaguing the Gauteng Department of Health [33].

There are limitations to this study. Some of the participants were known to the researcher and could have resulted in bias. Not all participants spoke English as a first language so interpretations could have been incorrect. Due to the nature of qualitative data there is always a risk of subjectivity. As a result, what actually happened may have been different to what was reported. The demographic data demonstrates that the participants had numerous years of experience in the current system and may have felt jaded, affecting their responses. Snowball sampling may have introduced selection bias.

## Conclusion

There is currently no clear functional triage system to manage the sorting of patients in non-emergency PHC outpatient settings across Gauteng’s CHCs, as perceived by clinicians interviewed. Based on the findings of this study and international literature it is recommended that a standardised protocol be developed when introducing a triage system into the primary health care setting [33]. The Ideal Clinic guideline recommendations of three hours as a maximum waiting time; a dedicated area for monitoring vital signs; and a process that prioritises high risk patients should serve as a basis [20]. Findings from this study can guide the development of a functional triage system. The Emergency Triage, Assessment and Treatment (ETAT), modified for adult settings and non-clinician use, could add to this [34].

## Abbreviations

CHCs: Community Health Centres
COREQ: Consolidated criteria for reporting qualitative research
ETAT: Emergency Triage, Assessment and Treatment
IMCI: Integrated Management of Childhood Illnesses
PHC: Primary Health Care
SATS: South African Triage Score
